# Colormap augmentation: a novel method for cross-modality domain generalization

**DOI:** 10.1007/s11548-025-03559-y

**Published:** 2025-12-15

**Authors:** Falko Heitzer, Duc Duy Pham, Wojciech Kowalczyk, Marcus Jäger, Josef Pauli

**Affiliations:** 1 Chair of Orthopaedics and Trauma Surgery, University of Duisburg-Essen, Essen, Germany; 2 Department of Orthopaedics, Trauma and Reconstructive Surgery, St. Marien-Hospital Mülheim, Mülheim, Germany; 3 Chair of Mechanics and Robotics, University of Duisburg-Essen, Duisburg, Germany; 4 Chair for Intelligent Systems, University of Duisburg-Essen, Duisburg, Germany

**Keywords:** Cross-modality, Data augmentation, Deep learning, Domain generalization, Intensity augmentation

## Abstract

**Purpose:**

Domain generalization plays a crucial role in analyzing medical images from diverse clinics, scanner vendors, and imaging modalities. Existing methods often require substantial computational resources to train a highly generalized segmentation network, presenting challenges in terms of both availability and cost. The goal of this work is to evaluate a novel, yet simple and effective method for enhancing the generalization of deep learning models in segmentation across varying modalities.

**Methods:**

Eight augmentation methods will be applied individually to a source domain dataset in order to generalize deep learning models. These models will then be tested on completely unseen target domain datasets from a different imaging modality and compared against a lower baseline model. By leveraging standard augmentation techniques, extensive intensity augmentations, and carefully chosen color transformations, we aim to address the domain shift problem, particularly in the cross-modality setting.

**Results:**

Our novel CmapAug method, when combined with standard augmentation techniques, resulted in a substantial improvement in the Dice Score, outperforming the baseline. While the baseline struggled to segment the liver structure in some test cases, our selective combination of augmentation methods achieved Dice scores as high as 83.2%.

**Conclusion:**

Our results highlight the general effectiveness of the tested augmentation methods in addressing domain generalization and mitigating the domain shift problem caused by differences in imaging modalities between the source and target domains. The proposed augmentation strategy offers a simple yet powerful solution to this challenge, with significant potential in clinical scenarios where annotated data from the target domain are limited or unavailable.

## Introduction

Automation of deep learning (DL)-based organ segmentation plays a crucial role in medical diagnostics and treatment planning, opening up new possibilities for evidence-based and personalized healthcare. Successful segmentation of specific structures depends on well-trained deep learning models. However, the training of such models requires large datasets with sufficient annotation and variability to ensure generalization from training to real-world application. Unfortunately, in clinical practice, access to well-annotated datasets is often limited, and relevant factors such as patient management, image acquisition, and manual annotation of medical images require a considerable amount of costs, time, and effort [1]. This becomes especially challenging when segmentations across different imaging modalities are needed. In such cases, a DL model would require a separate large training dataset for each distinct application area and domain [2].

The divergence between patients, clinics, scanner vendors, acquisition protocols, and even imaging modalities describes the ‘domain shift’ problem, which is a common challenge in clinical practice. The extent of this domain shift varies depending on the nature and range of differences between the domains [3]. Deep learning models trained on a specific dataset from one imaging modality (e.g., MRI) often struggle to perform well when applied to a dataset from a different modality (e.g., CT) [4]. This ‘cross-modality’ task is a very challenging aspect of generalizing to unseen domains, given the diverse physical processes underlying each imaging modality. The varying intensity-based representations of different modalities, combined with patient-specific morphological differences, significantly influence the magnitude of the domain shift [5].

There are several approaches to handle this domain shift problem, which are well described in the review by Garcea et al. [6]. A very new method, which requires additional information in the form of the location of a relevant structure (as a bounding box), is presented in the work of Ma et al. [7]. The so-called MedSAM method finds connected structures in the specified bounding box. However, it should be noted that the model was trained with a huge dataset (1570263 medical images) from 10 different modalities. In the domain generalization approach, on the other hand, the DL model should be trained and generalized without data from the target domain, such that the target domain data remains completely unknown [4, 8, 9]. A new method was recently published by Cai et al. [10]. Here, a Multi-task Adaptive Learner (MAL) is used to achieve generalizability for unknown domains. Even though the target domain is still unknown in this case, it should be noted that five data sets with different modalities were used for training, so that modality-specific differences are already known. A very recent approach that specifically addresses the problem of single-source domain generalization for cross-modality tasks was published by Chen et al. [11]. To use this method, large-scale pre-trained stable diffusion (SD) models are required so that diverse, realistic images can be generated. This approach is comparable to that of Jackson et al. [12], who perform a type of data augmentation by transferring styles and creating different images.

Data augmentation represents an important possibility to increase the variance in training data and to generalize better to unseen domains. Augmentation has, however, mostly been applied for rather small domain shifts, such as shifts caused by different patients or by different acquisition protocols within the same modality. It remains questionable whether these methods are sufficient for larger domain shifts, such as cross-modality generalization settings. Therefore, the main contributions of this work are:

1. Application of the methods BigAug [13] and style augmentation [12, 14] as specifically comparable methods for domain generalization in the **cross-modality** setting.
2. Introduction of a new, fast, and more efficient data augmentation method **CmapAug**, using colormap transformations.
3. Investigation of both **multi-source** and **single-source** domain generalization to cross-modality target domains.

## Related work

### BigAug

BigAug is a sequence of *n* stacked transformations $\tau$(.), as formulated in Eq. 1, where each transformation is an image processing function, and each function is associated with two parameters: 1) the probability *p* to apply the function and 2) the magnitude *m* of the function. Given training data $x_{s}$ and associated annotation $y_{s}$ , augmented data $\hat{x_{s}}$ and corresponding annotation $\hat{y_{s}}$ could be generated after *n* transformations through Eq. 1 [13]:

1 $$\begin{aligned} (\hat{x_{s}}, \hat{y_{s}})= \tau _{p_{n},m_{n}}^n (\tau _{p_{n-1},m_{n-1}}^{n-1} (... \tau _{p_{1},m_{1}}^1 (x_s,y_s))) \end{aligned}$$

This leads to an artificial enlargement of the training dataset. The publication of Zhang et al. [13] has shown a good generalization for DL models using the BigAug strategy. The models are trained on one dataset and tested on an unseen dataset from the same modality. In our case, we use the BigAug strategy as a benchmark method for domain generalization following [15].

### StyleAug

The style augmentation method can be categorized as intensity augmentation, where the texture, contrast, and color are modified. The style transfer pipeline published by Jackson et al. [12] was applied by Hesse et al. [14] in the medical area for image segmentation for the first time. Hesse et al. use style augmentation to segment breast structures in different MRI acquisition protocols.

The style transfer pipeline used by Hesse et al. [14] is based on a style transfer network and a style prediction network, which represents a pre-trained Inception-v3 network. The style prediction network extracts the style from the input image ($S_{predicted}$) and combines it with a weighted (weighting factor $\alpha$) random style embedding ($S_{random\,Embedding}$). The random style embedding is derived from a multivariate normal distribution whose mean and covariance are taken from the Painter By Numbers (PBN) data set. The resulting style is used as further input (together with the initial input image) for the style transfer network, which is pre-trained with the PBN data set and uses multiple convolutional layers in combination with a conditional instance normalization to adjust the initial input image. The resulting image ($S_{random\,+\,predicted}$) is generated using the formula (2):

2 $$\begin{aligned}&S_{random\,+\,predicted} \nonumber \\&\quad = \alpha \cdot S_{random\,Embedding} + (1 - \alpha ) \cdot S_{predicted} \end{aligned}$$

Due to the extreme change in intensity representation (texture, contrast, and color) by the style augmentation, image differences based on the various MRI acquisition protocols can be increasingly neglected, resulting in improved performance. In this work, we go one step further and apply style augmentation in a cross-modality setting to test the ability to handle a larger domain shift caused by a completely different imaging modality.

### Structure augmentation

The use of further relevant information has already been shown in the publication by Jiang and Gu [8], Pham et al. [16] and Su et al. [9]. In the publication of Su et al., a method called Saliency-balancing Location-scale Augmentation (SLAug) is used to extract local location information from the input mask and combine them with global location information from the input image. The method of Pham et al. follows a similar approach. By directly adding contour information during training and application, a significant improvement in segmentation could be achieved. Jiang and Gu also use an edge-guided method for the case of single-source domain generalization (EGSDG) in cross-sequence, cross-center, and cross-modality segmentation, respectively. Based on these results, a new method for intensity augmentation is explored in this work.

## Methods

### Segmentation network

**Fig. 1:**
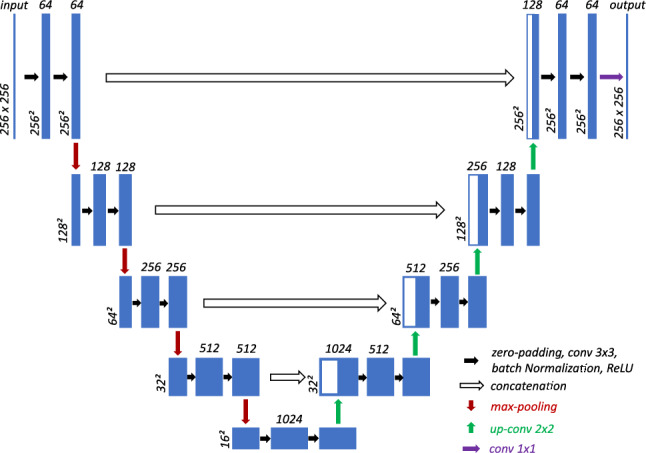
Segmentation network structure

The base network used in this work for segmentation of biomedical images is a 2D U-Net from Ronneberger et al. [17], which has been slightly adapted by zero padding and batch normalization. The architecture consists of 5 stages, in each of which 2 successive convolution operations (3x3 kernels) are performed. In every stage, a rectified linear unit (ReLU) activation function and batch normalization are used [18]. Since the convolution leads to a reduction of the tensor size, an additional zero padding is added. The structure, number of feature maps, image size, and applied operations in every stage of the down- and up-sampling path are shown in Fig. 1. The dice loss is used as loss function. For the optimizer, the adaptive moment estimation (Adam) method with an initial learning rate of $10^{-4}$ is used.

### Augmentation strategies

For generalization of the DL network, data augmentation is randomly performed before each training epoch to avoid overfitting. For the augmentation process, the following 4 approaches are used and combined with each other:

#### BigAug

The BigAug approach by Zhang et al. [13] is used as an approach for basic data augmentation. The approach is implemented with the albumentations and scikit-image libraries.

#### Extensive Augmentation (ExAug)

To extend and adapt the previously described approach, the ExAug approach is created. The application order, application strength, and probability are shown in Table 1. In order to ensure a realistic adjustment and an extensive diversity of the augmented data, all transformation ranges (minimum and maximum) of the BigAug approach were analyzed and adjustments were made on this basis. Furthermore, the ExAug approach was optimized for the combination with intensity-based augmentations. This approach was also implemented using the albumentations and scikit-image libraries.

**Table 1:** ExAug transformation with application strength and probability, listed in application order (No. 1 to No. 14)

**No.**	**Transformation**	**Strength**			**Probability**
1	CenterCrop and	200	–	220	50 %
	Resize ($256 \times 256$)				
2	Rotation	–10	–	10	50 %
3	Affine scale	0.8	–	1.2	50 %
4	Affine translate$\_$px	5	–	10	50 %
5	OpticalDistortion				50 %
	distort$\_$limit	$-$0.5	–	5	
	shift$\_$limit	0			
6	ElasticTransform				50 %
	alpha	4			
	sigma	2			
	alpha$\_$affine	−2	–	2	
	OneOf: No.7 - 10				50 %
7	Sharpen	0.1	–	0.3	30 %
8	GaussianBlur	0.25	–	1.5	20 %
9	GaussianNoise	0.005	–	0.01	30 %
10	MotionBlur	3	–	7	20 %
11	Intensity Perturbation	$-$0.1	–	0.1	50 %
	OneOf: No.12 - 14				50 %
12	RandomBrightnessContrast				50 %
	brightness$\_$limit	$-$0.1	–	0.1	
	contrast$\_$limit		0		
13	RandomGamma	50	–	150	50 %
14	ColorJitter				50 %
	brightness	0.6	–	1.5	
	contrast	0.6	–	1.5	
	saturation	0.6	–	1.5	
	hue	$-$0.25	–	0.25	

#### StyleAug

For intensity augmentation, the pipeline of Hesse et al. [14] with the style transfer algorithm of Jackson et al. [12] was adopted. The associated specifications were adopted (probability = 50 %, weighting factor $\alpha$ = 0.5 to control the strength of the style embedding). The pipeline was applied as described in section 2.2. For the input, 1 channel layered images were expanded to 3 channels by copying the first channel. Each slice was stylized individually. As in Hesse et al., the stylized output images were transformed back into 1 channel images using the PyTorch grayscale transformation.

#### Colormap Augmentation (CmapAug)

**Fig. 2:**
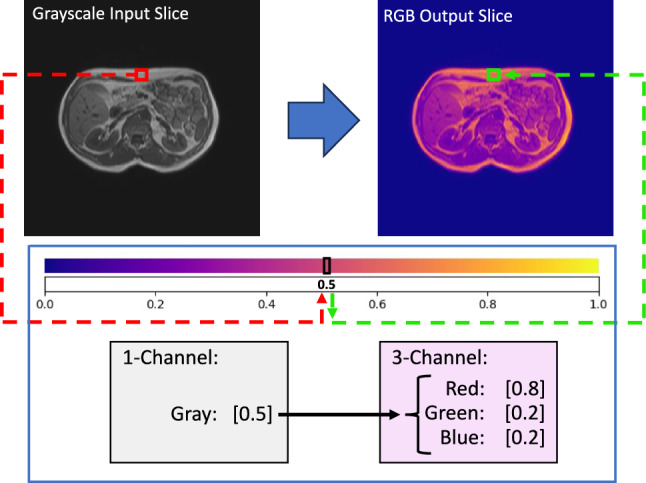
Colormap transformation method

As an alternative to the style augmentation, a new, more efficient method is proposed and investigated. Although a preliminary variant for 3D cases has been shortly discussed in previous work [19], to the best of our knowledge such a method has not yet been published in any peer-reviewed paper with this degree of analysis. By a simple colormap transformation, the grayscale images are transformed into RGB images. In our work, colormaps are used to align the DL model to abstract from intensity-based features and obtain more structural contextual information. By performing the complete model building and analysis process in the colorspace (model training, validation, and testing), modality-specific differences of the various imaging methods can be neglected and an improvement of the segmentation accuracy in the cross-modality scenario can be achieved. With predefined colormaps from Matplotlib, a specific color value is assigned to each gray value intensity. As shown in Fig. 2, no additional calculation is needed for this process, so the gray value is simply assigned to a specific RGB value, given by the chosen colormap.

**Fig. 3:**
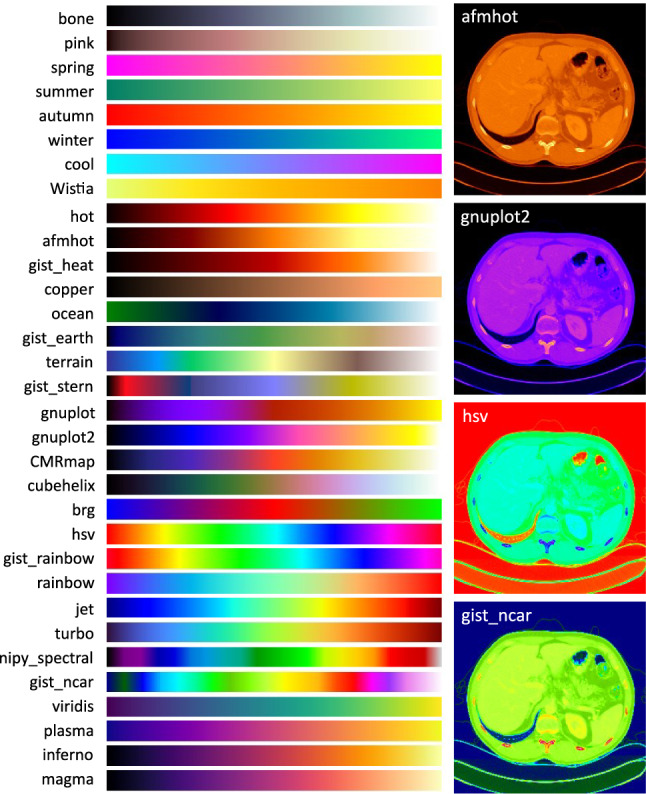
Selected colormaps and exemplary transformed CT slices

For augmentation, a colormap package of 32 different colormaps is manually selected. The preselection of the colormap package is based on two steps. First, colormaps that lead to a loss of image information due to the specific color code (assignment of multiple gray values to the same color values) were removed. This applies to all qualitative colormaps, as well as the miscellaneous colormaps ‘flag’ and ‘prism.’ Next, all colormaps were applied to the image data, to check the extent to which the data were adjusted. Based on this, the colormaps that generated minor changes in the image data were removed. This applies to all diverging colormaps, the cyclic colormaps ‘twilight’ and ‘twilight_shifted,’ all sequential colormaps, the perceptually uniform sequential colormap ‘cividis,’ and the sequential2 colormaps ‘binary,’ ‘gist_yarg,’ ‘gist_gray,’ and ‘gray.’ Figure 3 shows the color chart of all 32 colormaps and 4 exemplary transformed CT slices. One of the 32 colormaps is randomly selected for each input image during training. Due to its simplicity, it is not necessary to use GPUs for the colormap transformation, as opposed to style transformations. The probability of augmentation is set to 100 %. Through this, the training is performed completely in the RGB space. To reduce the gap between training and testing data, the validation and testing data are also transferred into the RGB space. For this purpose, a predefined colormap of the 32 maps is used for the whole validation and test data, so that all these images are processed under the same conditions. The selection of this colormap is based on experiments with each colormap individually. For these experiments, a U-Net was trained with all 32 maps for augmentation, validated with one specific colormap after each training epoch, and tested with the same colormap. The colormap with the best performance was chosen (gnuplot2, Dice Score: 83.1 %). The random transformation of the input images should lead to a continuous change of the image intensities, so that only the image structures are learned.

#### Combination of approaches

Since the two augmentation approaches BigAug and ExAug and the two augmentation approaches StyleAug and CmapAug are, respectively, similar in their structure and application goal, one of each pair is combined with each other. Considering also the 4 augmentation approaches in an individual manner, a total of 8 augmentation strategies will be investigated as ablation studies.

### Datasets

Four different datasets from multiple vendors and scanners were used to evaluate the various data augmentation approaches. The main publicly available datasets are from the ‘Combined Healthy Abdominal Organ Segmentation (CHAOS) Challenge’ [20, 21]. The CHAOS datasets can be divided into an MRI (20 healthy patients, a total of 1294 T1, and 623 T2 slices with a resolution of 256x256) and a CT dataset (20 healthy patients, a total of 2874 slices with a resolution of 512x512). For both datasets, images were acquired from different patients. For training the DL network, only one of the two datasets is used (source domain). Testing is performed on the other dataset (target domain). To verify these results, two additional publicly available CT datasets are used. One dataset comes from the database ‘The Cancer Imaging Archive (TCIA)’ [22] (43 healthy patients, a total of 10235 slices with a resolution of 512x512) and the other dataset from the ‘Beyond the Cranial Vault (BTCV)’ challenge [23] (47 cancer or hernia studies patients without tumor in the liver, a total of 5774 slices with a resolution of 512x512). These datasets are only used to test the model (target domain) when trained with MRI data. For all datasets, the ground truth mask of the liver is available.

### Preprocessing

In the first step, all outliers in the CT data were eliminated. For this purpose, all intensity values exceeding the value range of the Hounsfield scale [24] were set to the volume’s maximum value within the value range. Subsequently, all image slices (CT and MRI) were set to the value range 0 to 1 using the min-max normalization. As the last step, all input data slices (training, validation, testing) were resized to 256x256, so that all used datasets from different scanners and vendors have the same image size.

### Evaluation metrics

Two established metrics are used for evaluation, one of which is widely used as main metric (Dice score) [8, 9, 14, 16]. As the Dice score measures segmentation performance only by the overlap ratio between prediction and ground truth, the Hausdorff distance is additionally used to indicate the magnitude of the deviations and thus a measure of the incorrect classifications. During the validation process of each training epoch, the Dice score is used to calculate the compliance of each pixel between the prediction and ground truth for every single image (2D) [25]. For the evaluation, the Dice score and the Gromov–Hausdorff metric (Hausdorff distance) were used. The Hausdorff distance calculates the maximum deviation between the prediction and the ground truth [26]. During the evaluation (testing), the Dice score and the Hausdorff distance are calculated for each patient volume (3D).

## Experiments and results

All DL networks were trained and tested on a GeForce RTX 2080 SUPER (8 GByte memory) with a batch size of 12 for training. For better comparability, the same seed points were set for all cases during initialization. By using PyTorch as the main library, the networks were trained for 200 epochs (MRI data) or 50 epochs (CT data), due to the higher amount of slices per patient volume in CT data.

For the evaluation of the different augmentation strategies, both multi-source and single-source domain generalization scenarios were considered. In the case of multi-source domain generalization, MRI images from both T1- and T2-weighted protocols were used for training, with generalization to CT data (case 1). For single-source domain generalization, CT images were used for training, with the model being generalized to either T1- or T2-weighted MRI sequences (case 2). Table 2 shows the partitioning of the datasets for both cases.

**Table 2:** Experiment cases for evaluation, distribution of source, and target domain data

**Case**	Source Domain	Images	Target Domain	Images
**1.**	CHAOS MRI		CHAOS CT	2874
	T1 + T2	1917	BTCV	5774
			TCIA	10235
**2.**	CHAOS CT	2874	CHAOS MRI	1294
			(T1 or T2)	623

**Fig. 4:**
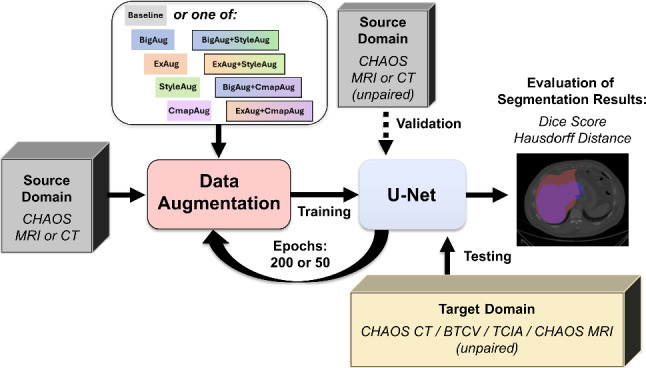
Flowchart of the experiments

To illustrate the structure and sequence of the experiments, the general flowchart is shown in Fig. 4. In the training process, the source domain data are divided into two parts, so that 3/4 of the patients are used for the actual training and 1/4 of the patients are used for the validation of each training epoch. This ensures that only data from the source domain are used to optimize the hyperparameters. The training process is based on 2D slice data to ensure a larger training dataset. The selection of the best model is based on the highest Dice score, which is calculated in 2D after each training epoch for the validation data. The target domain data remain completely unseen. The results of the testing process are calculated in 3D by each patient volume.

**Fig. 5:**
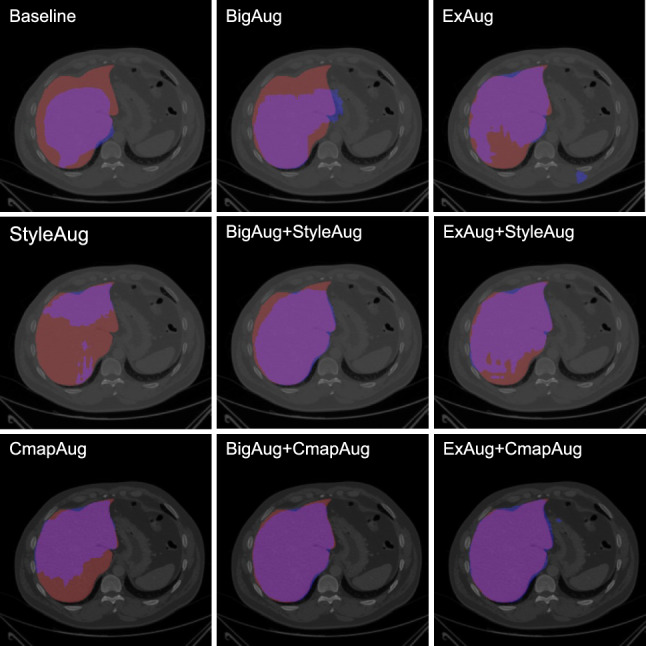
CHAOS CT slice for each augmentation strategy with segmented liver structure (ground truth: red = false negative; predicted segmentation: blue = false positive; and overlay: purple = true positive)

The baseline results are presented together with the results of all 8 different augmentation strategies in Tables 3 - 4. An exemplary slice image is shown in Fig. 5, where the same slice is shown for each augmentation strategy.

**Table 3:** Test results case 1 (source domain = MRI T1 + T2, target domain = CT)

Case 1	Dice Score [%] (Std.)			Hausdorff Distance [Voxel] (Std.)		
Augmentation strategies	CHAOS CT	BTCV	TCIA	CHAOS CT	BTCV	TCIA
Baseline	56.2 (8.3)	33.4 (11.6)	56.7 (9.0)	122.4 (33.5)	135.5 (25.6)	144.6 (22.2)
BigAug	68.2 (14.1)	63.3 (10.6)	65.4 (14.1)	102.7 (28.6)	115.8 (29.8)	106.8 (28.8)
ExAug	75.5 (14.0)	67.8 (11.8)	70.1 (13.2)	107.3 (29.1)	131.2 (25.8)	114.3 (26.4)
StyleAug	79.5 (17.5)	76.2 (14.5)	79.5 (11.3)	52.1 (29.67)	**71.5 (34.7)**	**46.7 (29.2)**
CmapAug	83.1 (17.6)	68.9 (19.4)	81.0 (13.5)	44.7 (23.2)	95.4 (35.0)	65.6 (34.9)
BigAug+StyleAug	84.2 (10.7)	79.4 (6.7)	78.8 (10.0)	**38.7 (24.6)**	76.4 (28.6)	53.5 (26.4)
ExAug+StyleAug	86.4 (10.0)	83.2 (5.9)	85.3 (6.3)	54.3 (36.1)	111.9 (33.8)	55.1 (34.1)
BigAug+CmapAug	87.3 (9.8)	74.1 (8.7)	83.9 (6.7)	52.2 (31.5)	95.1 (32.6)	68.1 (28.2)
ExAug+CmapAug	**89.5 (11.1)**	**85.9 (6.0)**	**88.3 (3.8)**	40.5 (28.4)	89.5 (32.2)	52.6 (32.0)

**Table 4:** Test results case 2 (source domain = CT, target domain = MRI T1 or T2)

Case 2	Dice Score [%] (Std.)		Hausdorff Distance [Voxel] (Std.)	
Augmentation strategies	CHAOS MRI T1	CHAOS MRI T2	CHAOS MRI T1	CHAOS MRI T2
Baseline	0 (0)	0 (0)	119.2 (63.6)	324.4 (96.0)
BigAug	71.0 (13.0)	72.2 (17.9)	**25.7 (8.7)**	**28.8 (16.1)**
ExAug	50.6 (19.6)	36.8 (15.9)	49.3 (19.4)	42.4 (26.7)
StyleAug	65.9 (15.1)	2.8 (4.3)	40.3 (15.9)	71.1 (27.1)
CmapAug	28.9 (20.8)	0.5 (1.9)	44.5 (14.6)	155.3 (125.4)
BigAug+StyleAug	80.3 (7.1)	**78.5 (12.7)**	29.2 (17.7)	29.2 (19.4)
ExAug+StyleAug	68.6 (12.7)	48.6 (17.2)	29.6 (15.2)	68.4 (18.5)
BigAug+CmapAug	75.7 (8.7)	64.6 (9.3)	33.5 (15.5)	28.9 (15.0)
ExAug+CmapAug	**83.2 (5.1)**	41.0 (12.1)	26.2 (13.3)	30.5 (13.7)

Table 4 (case 2) shows that the existing domain shift between the different modalities is too large for the baseline model. The newly developed CmapAug approach in combination with the ExAug approach leads to the best Dice score for all 3 unseen target domain datasets in the multi-source domain generalization experiment (Table 3). The combined augmentation strategy ExAug+CmapAug achieved an enormous improvement compared to the lower baseline (Table 3: Dice score min. +31.6%, max. +52.5%; Table 4: Dice score min. +41.0%, max. +83.2%), although no complex calculations are performed. This could be related to the implementation of the ExAug and CmapAug approaches. Compared to the BigAug approach, the ExAug approach uses more transformations with a lower intensity and a different stacking (Table 1). The CmapAug approach in comparison with the StyleAug approach leads to minor changes in texture and a more consistent color transformation.

However, it should be pointed out that in the application on the target domain MRI T2 BigAug+StyleAug achieved a bigger improvement than ExAug+CmapAug and the abstraction from intensity-based features with the individual approaches StyleAug or CmapAug achieved no considerable improvements. This may result from the fact that the representation of the structures in the MRI T2 image is extremely dark, so that the StyleAug and CmapAug methods alone do not produce enough modifications. Nevertheless, the combination of ExAug and CmapAug also achieved a considerable improvement and in most cases the best Dice score, making it a well-balanced augmentation strategy to apply standard augmentations together with a simple and resource-efficient color transformation.

## Discussion

Our results show that the considered augmentation strategies can also be used to address the domain shift problem in cross-modality settings. Not only do the chosen benchmark augmentation techniques lead to an improvement of the segmentation, but also the novel colormap augmentation CmapAug can be used for this purpose. Although we only use a 2D U-Net and no further backbone, as it is done in comparable publications [9], promising results have been achieved. This shows that our CmapAug method has high potential in combination with standard augmentation methods (e.g., BigAug, ExAug) and established model setups (e.g., 3D segmentation network, pre-trained backbone). A clear limitation of our study is the focus on 2D data, which should be extended to 3D studies in further research, as it was done in [19]. Furthermore, we used an almost unmodified raw segmentation network (U-Net) [17] whose performance could be improved by backbones as used in other publications [9].

## Conclusion

In this paper, several augmentation strategies have been used for domain generalization in the challenging cross-modality setting, considering both multi-source and single-source domain generalization. In this context, a new and efficient colormap-based intensity augmentation method has been proposed and investigated. Colormap augmentation is a very simple and efficient way to improve segmentation in different domains. Without the use of GPUs or pre-trained style transfer networks, data from an unknown domain can be segmented. The experiments show that in combination with standard augmentation techniques, very promising improvements in comparison with the baseline and the chosen benchmark methods can be achieved. Even in the case of training with only one modality, a sufficiently good segmentation is observed. However, the application of this method to other organs, pathological tissue (e.g., tumors), and different modalities is also possible and should be able to improve the segmentation performance in further domain generalization tasks.

## Declarations

### Source code

The source code is available at: https://github.com/dp-at-ude/colormap_augmentation
